# Correction: A novel fully conjugated COF adorned on 3D-G to boost the “D–π–A” electron regulation in oxygen catalysis performance

**DOI:** 10.1039/d5sc90281a

**Published:** 2026-01-06

**Authors:** Yinggang Sun, Wenjie Duan, Jigang Wang, Peng Sun, Yanqiong Zhuang, Zhongfang Li

**Affiliations:** a College of Chemistry and Chemical Engineering, Shandong University of Technology Zibo 255000 Shandong P. R. China zhfli@sdut.edu.cn

## Abstract

Correction for ‘A novel fully conjugated COF adorned on 3D-G to boost the “D–π–A” electron regulation in oxygen catalysis performance’ by Yinggang Sun *et al.*, *Chem. Sci.*, 2025, **16**, 9951–9965, https://doi.org/10.1039/D5SC02082D.

The authors regret that the institution name in the author affiliations was incorrect in the original article. The correct institution name (Shandong University of Technology) and information is as shown herein.

The Royal Society of Chemistry apologises for these errors and any consequent inconvenience to authors and readers.

